# The Effects of a Problem Solving-Based Intervention on Depressive Symptoms and HIV Medication Adherence Are Independent

**DOI:** 10.1371/journal.pone.0084952

**Published:** 2014-01-06

**Authors:** Robert Gross, Scarlett L. Bellamy, Jennifer Chapman, Xiaoyan Han, Jacqueline O’Duor, Brian L. Strom, Peter S. Houts, Steven C. Palmer, James C. Coyne

**Affiliations:** 1 Center for Clinical Epidemiology and Biostatistics and Department of Biostatistics and Epidemiology, University of Pennsylvania Perelman School of Medicine, Philadelphia, Pennsylvania, United States of America; 2 Center for Pharmacoepidemiology Research and Training, University of Pennsylvania Perelman School of Medicine, Philadelphia, Pennsylvania, United States of America; 3 Division of Infectious Diseases, Department of Medicine, University of Pennsylvania Perelman School of Medicine, Philadelphia, Pennsylvania, United States of America; 4 Philadelphia Veterans Affairs Medical Center, Philadelphia, Pennsylvania, United States of America; 5 Pennsylvania State University College of Medicine, Hershey, Pennsylvania, United States of America; 6 Abramson Cancer Center, University of Pennsylvania Perelman School of Medicine, Philadelphia, Pennsylvania, United States of America; 7 Health Psychology Section, Department of Health Sciences, University Medical Center, University of Groningen, Groningen, the Netherlands; Massachusetts General Hospital, United States of America

## Abstract

Depression and depressive symptoms predict poor adherence to medical therapy, but the association is complex, nonspecific, and difficult to interpret. Understanding this association may help to identify the mechanism explaining the results of interventions that improve both medical therapy adherence and depressive symptoms as well as determine the importance of targeting depression in adherence interventions. We previously demonstrated that Managed Problem Solving (MAPS) focused on HIV medication adherence improved adherence and viral load in patients initiating a new antiretroviral regimen. Here, we assessed whether MAPS improved depressive symptoms and in turn, whether changes in depressive symptoms mediated changes in adherence and treatment outcomes. We compared MAPS to usual care with respect to presence of depressive symptoms during the trial using logistic regression. We then assessed whether MAPS’ effect on depressive symptoms mediated the relationship between MAPS and adherence and virologic outcomes using linear and logistic regression, respectively. Mediation was defined by the disappearance of the mathematical association between MAPS and the outcomes when the proposed mediator was included in regression models. Although MAPS participants had a lower rate of depressive symptoms (OR = 0.45, 95% confidence interval 0.21–0.93), there was no evidence of mediation of the effects of MAPS on adherence and virological outcome by improvements in depression. Thus, interventions for medication adherence may not need to address depressive symptoms in order to impact both adherence and depression; this remains to be confirmed, however, in other data.

## Introduction

Adherence to antiretroviral drugs is central to HIV treatment success, with the goal being suppression of plasma virus levels to below the limit of quantification (i.e., “undetectable viral load”). Yet, non-adherence to medical therapy occurs in upward of 50% of patients on efficacious self-administered therapies [1], resulting in treatment failure and avoidable complications of disease. Depressive symptoms are common among HIV infected individuals [2], and a recent meta-analysis showed that depressive symptoms are a marker for poor treatment adherence [3]. Moreover, the association was not limited to major depressive disorder, but extended to subclinical levels of depressive symptoms [4], leading authors of that meta-analysis to call for interventions that focus on reducing severity of depressive symptoms even when symptoms are at subclinical levels.

A limited literature suggests that treatment targeting both depressive disorders and adherence with psychotherapy improves adherence to HIV treatment [5]. For instance, a recent study showed that Cognitive Behavioral Therapy for Adherence and Depression in HIV-infected injection drug users improved both depressive symptoms and adherence, but not viral loads [6]. However, causal interpretations of the effect of treating depression on adherence outcomes are complicated by consistent findings that depressive symptoms are correlated with a full range of other negative affect variables, as well physical health problems, concurrent stress, and adjustment problems [7]. Many of these factors are also barriers to medication adherence. Therefore, addressing these factors directly, rather than addressing depression, may be a more direct route to improving adherence.

The pragmatic clinical issue remains whether we can afford not to address depressive symptoms in interventions focused on adherence and still obtain the effect on medication taking. If depressive symptoms are a marker for poor adherence, do improvements in depressive symptoms serve as a mediator for improved adherence? Regardless, can a secondary reduction in depressive symptoms be a basis for further recommending interventions that improve adherence? Answers to these questions have important implications for the design and dissemination of interventions to improve adherence.

We addressed these questions by taking advantage of data from a previously described [8] clinical trial of an HIV adherence intervention, Managed Problem Solving (MAPS). MAPS resulted in an average increase of 15.2% of doses taken (p<0.001) and a 1.48 (95% CI: 0.94–2.31) times greater odds for undetectable viral load compared to usual care [8]. This face-to-face problem solving-based counseling intervention is particularly relevant to the question of whether reductions in depressive symptoms mediate adherence effects because it is derived from problem-solving therapy for depression, but it is intended to address only specific barriers to adherence, not depression. The MAPS treatment protocol (available at http://www.med.upenn.edu/cceb/maps-form.shtml) specifically excludes any focus on general problem orientation or expectancies (i.e., those problems or expectancies beyond the focus on adherence to antiretroviral therapy or HIV treatment outcomes) that are considered so crucial in problem-solving therapy for depression [9]. In MAPS, when possible, clinical depression is identified as one of the barriers to adherence, referrals are simply offered for formal diagnosis and treatment outside of the intervention.

In the present study, we aimed to determine whether MAPS improved depressive symptoms, and if so, whether improvement in depressive symptoms mediated the effect of MAPS on adherence and virologic response, taking into account the offering of referral for depression treatment.

## Methods

We conducted a secondary analysis of a randomized clinical trial comparing MAPS to usual outpatient HIV care in HIV infected individuals newly starting or changing antiretroviral regimens due to prior treatment failure or discontinuation. Details of the MAPS trial have been previously published [8]. Briefly, we recruited 180 patients (91 MAPS, 89 usual care) from 3 HIV clinics in Philadelphia via word of mouth from clinicians and pharmacists and fliers sent directly to patients. Usual care at the sites included meeting with a pharmacist for education about the regimen, potential side effects, and if desired, provision of pill organizers. Exclusion criteria were inability to provide informed consent and residence in a setting where medications were delivered automatically.

### Delivery of MAPS Intervention

Three interventionists were trained over 15 hours each regarding HIV disease and treatment, depression and substance abuse, and in the use of the Managed Problem Solving treatment manual and workbooks. Interventionists were required to have a college degree and experience working with patients.

The MAPS intervention includes 5-steps: 1)identifying participant’s barriers to adherence, 2)brainstorming for potential solutions, 3)selecting the best option, 4)monitoring whether it was implemented and considered useful, and 5)monitoring the participant’ s adherence and reiterating steps 2–4 if solutions were either not implemented or unsuccessful. Notably, the problem solving strategy was designed to overcome barriers to adherence, not to improve a patient’s problem solving skills as a general means of improving either adherence or depression. Baseline screening for all participants was conducted by a single study coordinator who was not one of the interventionists. The screening aided in the identification of common potential adherence barriers, using CES-D for depressive symptoms [10], the AUDIT [11], and ASI questionnaires [12] for substance abuse, and a questionnaire regarding HIV knowledge, health, and religious beliefs. If CES-D scores were ≥22, participants were then asked about duration of mood disturbance. If endorsed to be present for two weeks, depressive symptoms were identified as a potential adherence barrier. In the MAPS group, the interventionist focused the 5-step process on accessing depression counseling and/or pharmacotherapy, including a) referring the client for a psychiatric evaluation, b) referring the client to a counseling center or treatment program; c) asking to call their provider or having them speak to their provider; d) having them speak to clergy; e) enlisting the support of family or close friends, or f) offering the support of the interventionist, depending on the patients’ preferences.

We intended for the MAPS interventionists to deliver 4 face-to-face sessions and 12 telephone check-ins over the initial 3 months of the study and then 9 monthly refill reminder calls for the remainder of the year. Fidelity to the intervention was above the *a priori* designated threshold in all cases based on standardized evaluations of recorded sessions [8]. We targeted a sample size of 180 participants for at least 80% power to determine if MAPS improved adherence by at least 10% of doses.

### Measures

The CES-D was used to measure depressive symptoms quarterly for one year [10]. A cutoff of 22 was *a priori* designated to be consistent with depression rather than the usual cutoff of 16 because in a primary care setting, an elevated CES-D has moderate specificity, but low (<35%) positive predictive value, still necessitating a clinical inquiry to determine if treatable depression is present [13]. We also included CES-D score as a continuous measure in further analyses, because of concerns expressed in the literature that dichotomization loses information and increases the risk of Type II error [14], [15].

Adherence was measured over time using electronic monitors (MEMS, Aardex, Switzerland) and summarized as proportion of doses taken over each quarter for one year. Plasma HIV-1 RNA concentration was measured quarterly over 1 year with an assay having a lower limit of 75 copies/ml (Versant, HIV-1 RNA 3.0, Bayer Corp. Berkeley, CA).

We also assessed participants with CES-D scores ≥22 with respect to whether they sought treatment for depression including counseling and/or antidepressant medications.

### Analysis

The focus of these analyses was to compare the experimental and control groups for the presence of depressive symptoms over time and determine whether these symptoms mediated the effect of MAPS on adherence and virologic suppression. We used an intention-to-treat approach to compare the presence of depressive symptoms over time between the MAPS and usual care groups. We estimated the association between study group and differences in CES-D score and CES-D score≥22 over all four quarters of the year using generalized estimating equations (GEE) with linear regression and logistic regression for the respective outcomes [16]. Individuals who were lost to follow-up were included as having virologic failure at all subsequent time points [17]. In secondary analyses, we tested for potential confounding by measured variables by including covariates in the GEE models and inspecting for changes in the point estimate of the relation between study group and outcome. Potential confounders included baseline CES-D score≥22, race, sex, and age. We also tested for effect modification by time including an interaction term (MAPS x time point) in the models.

We assessed whether improvements in depressive symptoms mediated the effect of MAPS in separate GEE models for the outcomes of adherence and virologic suppression. We used the values from the primary analyses of the effect of MAPS on adherence as the base case. We included percent of doses taken over each quarter as a continuous variable and modeled the relation between MAPS and adherence using linear regression. We then added presence of high-level depressive symptoms for each participant at each time point into the models. We then compared the point estimate of the effect of MAPS on the outcome between the models with and without depression. We then repeated these analyses for the outcome of viral suppression, but using logistic regression instead of linear regression. We interpreted changes in the point estimate of the relation between the intervention and outcomes toward 1 as evidence of mediation. In addition, we assessed whether changes in depressive symptoms within the individual over time were associated with changes in that individual’s adherence over time. Specifically, we calculated the change in CES-D score over each interval and assessed the association with adherence in the subsequent interval using linear regression with GEE.

We then tested whether baseline elevated CES-D scores modified the effect of MAPS on later depressive symptoms by including an interaction term (intervention group x baseline CES-D>22) in the GEE models.

In order to determine the effect of MAPS on patients with high levels of depressive symptoms obtaining mental health care, we compared the proportion of individuals in the MAPS and usual care arms with CES-D scores>22 with respect to the proportion seeking treatment of depression and taking antidepressant medications during the course of the study using Chi-squared and Fisher’s exact tests.

### Ethical Considerations

We obtained written informed consent from all participants. The study was approved by the Committee on Human Subjects Research of the University of Pennsylvania and the Philadelphia Veterans Affairs Medical Center. Participants were compensated $30 per data collection visit, but not intervention visits. There were no early stopping rules. The ClinicalTrials.gov number is NCT00130273.

## Results

Depressive symptoms were common with 41 participants meeting the threshold of CESD≥22 at baseline; 19 (21%) in MAPS and 22 (25%) in usual care arms, respectively. Table 1 displays differences between those with and without baseline CES-D score≥22. Higher CES-D scores were associated with being female, hazardous alcohol use, being unemployed, and having low income, although none of these differences were statistically significant. MAPS was associated with a 2.3 point lower CES-D score across all time points (p = 0.046).

**Table 1:** Baseline Characteristics by Depressive Symptoms.

Characteristic	CESD≥22 (n = 41)	CESD<22 (n = 137)	p value
Study group			
MAPS	19 (21%)	72 (79%)	0.49
Usual Care	22 (26%)	65 (71%)	
Median Age (Interquartile range)	43 (39–49) years	43 (35–51)	>0.5
Female sex	21 (51%)	50 (37%)	0.09
Race			
Black	33 (80%)	118 (86%)	0.29
White	8 (20%)	16 (12%)	
Other	0	3 (2%)	
Income<$5000/yr	18 (44%)	39 (29%)	0.06
Hazardous Alcohol use	11 (27%)	21 (15%)	0.09
History of injection drug use	9 (22%)	20 (15%)	0.26
Currently employed	4 (10%)	31 (23%)	0.07
HIV Treatment naïve	19 (46%)	53 (39%)	0.38

Table 2 depicts the various models of the association between MAPS and high-level depressive symptoms (CES-D score≥22) at each quarter of follow-up. When time point was included as a potential confounder in the models, there was no change in the point estimate or 95% CI of the relation between MAPS and presence of high-level depressive symptoms. Further, there was no evidence of effect modification by time on the relation between MAPS and high-level depressive symptoms (MAPS x time point interaction term p value = 0.99). Although the 95% confidence intervals cross 1 at each time point, MAPS was associated with a lower proportion of participants with high-level depressive symptoms throughout the study. In the unadjusted model, MAPS was associated with a decreased odds of depressive symptoms over time. In addition, there was no association between change in depressive symptoms over each interval and change in adherence over each subsequent interval. For every 1 point increase in depressive symptoms over time, adherence decreased by 0.1% of doses taken, p = 0.4.

**Table 2:** Effect of MAPS on CESD Score>22 at Each Time Point.

Time Point	Odds Ratio (95% CI) for Presence of CESD Score≥22 in MAPS vs. UC
**Month 3**	0.46 (0.17–1.20)
**Month 6**	0.41 (0.13–1.31)
**Month 9**	0.42 (0.16–1.15)
**Month 12**	0.47 (0.16–1.37)

Table 3 displays the analyses assessing for potential confounders or mediators. Baseline CES-D score≥22 did not confound the protective effect of MAPS. In the mediation analyses, again, there was no effect of including either adherence or virologic suppression in the models. Nor was there evidence that the effect of MAPS on depressive symptoms differed between those with and without baseline CESD score≥22 (p value for interaction between MAPS and baseline CESD score = 0.45.

**Table 3:** Assessment of Potential Confounding or Mediation of the Relation between MAPS and CES-D Score≥22 Over Time.

Variables included in model	Odds Ratio (95% CI) for Presence of CESD Score≥22
MAPS vs. UC (Base case)	0.44 (0.21–0.93)
MAPS vs UC+Baseline CESD Score>22	0.50 (0.23–1.10)
MAPS vs. UC+Adherence over Time	0.55 (0.24–1.26)
MAPS vs. UC+Virologic Suppression over Time	0.50 (0.24–1.10)

There was no evidence that depressive symptoms mediated the effect of MAPS on either adherence (increase of 15.9% of doses taken, p = 0.0015) or virologic suppression (OR 1.72 (95% CI 1.00–3.00), with the point estimates remaining significant and not decreasing from the unadjusted models for these outcomes.

The proportion of individuals who sought counseling for depression was similar between the groups, 18/91 (20%) in the MAPS arm and 25/89 (28%) in the usual care arm (p = 0.19). The majority were seen by psychiatrists, 17 (94%) in MAPS and 21 (84%) in usual care (p = 0.38). Antidepressants were prescribed for 13 (72%) MAPS participants and 21 (84%) usual care participants (p = 0.35). These results suggest that MAPS did not have its effect on depressive symptoms merely by increasing the referral and treatment of depression by other providers.

## Discussion

We found that a problem-solving based intervention, MAPS, to be effective not only at improving adherence and virologic suppression as shown previously [8], but also at improving depressive symptoms. The improvement in depressive symptoms was not different over the different time points and was not confounded by any of the measured factors. The lack of association between change in depression symptoms and change in adherence over time and the lack of change in the point estimate of the association between MAPS and the outcomes when depression was included in the models both suggest that the effect of MAPS on adherence was not mediated by its effect on depression.

The lack of increase in use of services for depression by participants with high CES-D scores also suggests that specific MAPS activities did not directly impact receipt of care for depression. Further data indicate that among patients in regular contact with the medical system because of management of a life-threatening chronic condition, rates of prescription of antidepressants are high [18], [19], but quality of care for depression may be low because of subordination of the goal of treating depression to the competing demand for adequately managing the chronic illness [20]. Rather than leaving this complex issue to speculation, future research should ascertain the specificity with which antidepressants are prescribed to patients in treatment for HIV and the quality of depression care. The MAPS group received more in-person visits and telephone calls throughout the year than the control group, by nature of the intervention. These visits may have resulted in improvements in depressive symptoms, but these visits only occurred over the first 3 months of the study. The visits between the experimental and usual care subjects were equal over the remainder of the year. This added contact may have been responsible for the improvement in depressive symptoms, since referrals for treatment did not differ between the groups.

Observational studies have suggested that treatment of depression would result in improved treatment adherence[21]–[23]. However, a recent study of directly observed fluoxetine to treat depression in HIV resulted in improvements in depressive symptoms, but no change in HIV treatment outcomes. [24] Similarly, a trial of a cognitive behavioral therapy intervention for adherence and depression in HIV infected individuals resulted in improved adherence and depressive symptoms, yet only the improvement in depressive symptoms persisted after the intervention was discontinued [6]. The lack of association between improvements in depression and improvements in adherence in these two intervention studies targeting depression is consistent with our observation that improvement in depressive symptoms does not mediate improvement in adherence.

Even if MAPS does not directly address depressive symptoms, there may be important nonspecific elements of the interaction between MAPS interventionists and patients that serve to reduce these symptoms. The MAPS intervention involves regular contact with a supportive interventionist who inquires about patients’ management of their HIV regimen and collaborates in solving a problem the interventionist and patients negotiate. Interventionists were trained to focus on adherence barriers *per se*, but were also trained to build rapport by listening sympathetically to other issues participants raised. Because the interventionist has less of a history or external incentives for insisting upon patients’ adherence, the interventionist is in a position to be less blaming and critical than patients’ providers may become. Thus, they may positively influence adherence without the negative affective impact that more critical providers may engender. To test whether these speculations about nonspecific influences in the MAPS intervention are true, future trials might seek to accentuate supportive accountability [25], rather than attempting to control for it, and examine their secondary effect on depressive symptoms, as well as the primary outcomes of medication adherence and viral loads.

This study has several potential limitations. Although we measured depressive symptoms, we did not determine whether these symptoms represented a clinical diagnosis of depression or more diffuse distress. With the standard cutoff ≥16, the CES-D has been found to have a sensitivity of 79.5% and a specificity of 71.1% for major depression [26]. We used a higher cutoff of ≥22, which would increase the positive predictive value, but most patients with scores above this cut point would likely still not meet diagnostic criteria for clinical depression. Yet, the median CES-D score was quite high in this sample, increasing the likelihood that if depressive symptom improvement mediated the outcomes, we would have identified it. Although this was only a single-blinded study, the CES-D was administered by staff who were not part of the intervention team to decrease the likelihood of biased reporting of depressive symptoms. In general, receipt of a prescription for antidepressants was high in both the intervention and control group members with high CES-D scores, but we have no data concerning why patients chose their mode of mental health care, or the effectiveness, appropriateness, or quality of this treatment. We also did not assess use of services by patients with lower CES-D scores and therefore cannot comment on MAPS effect on seeking of mental health care by participants with lower levels of distress. Furthermore, as a real-world intervention, participants in the experimental arm did not universally adhere to the protocol [8]. Yet even despite imperfect implementation, the intervention still improved depressive symptoms. Thus, it is possible that we are underestimating the strength of the effect of MAPS on depressive symptoms.

This study has several strengths. It was conducted as a randomized trial, which controls for known and unknown confounders of the relation between the intervention and outcomes. Objective outcome measures were included-adherence via microelectronic monitors and treatment success via HIV viral loads. Further, depressive symptoms were followed serially over 1 year allowing assessment of the effect over time.

In conclusion, we found that a problem solving-based adherence intervention improved depressive symptoms, but did so independent of its effect on adherence and biological outcomes. Future modifications to adherence interventions such as MAPS may not need to be targeted to depressive symptoms to have an effect on adherence in a highly symptomatic population. This hypothesis warrants testing in further trials of medication adherence interventions.
